# Supplementary material for: A transcriptional switch controls sex determination in Plasmodium falciparum
Source: Nature. 2022 Dec 7;612(7940):528–33. doi: 10.1038/s41586-022-05509-z (PMC9750867; doi:10.1038/s41586-022-05509-z)
Supplement: Supplementary file 1 — Supplementary Figs. 1 (Flow cytometry gating strategies used in the study) and 2 (Raw gels for protein blots), and details of the files deposited on Zenodo. [file 41586_2022_5509_MOESM1_ESM.pdf]

nature portfolio

https://doi.org/10.1038/s41586-022-05509-z

Supplementary information

A transcriptional switch controls sex determination in *Plasmodium falciparum*

In the format provided by the authors and unedited

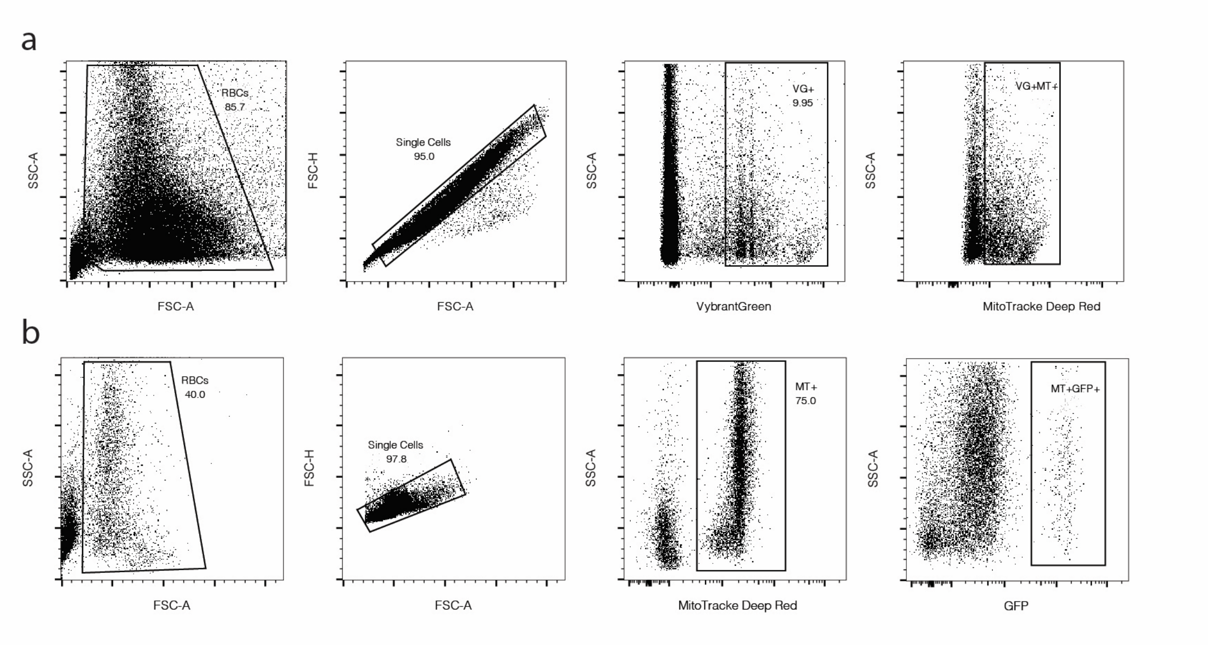

SI Figure S1. Gating strategies for flow cytometry. (a) Gating strategy used to determine gametocyte viability (Extended Data Fig 1e, Extended Data Fig 2f), cells are selected on size, single cells, and parasitized cells (VG+), viability is established on this population (VG+MT+). (**b)** Gating strategy used to sort cell for the Md1-2A-GFP dataset (Fig 3a), cells are selected on size, single cells, and parasitized cells (MT+), GFP + or – cells were sorted for scRNAseq.

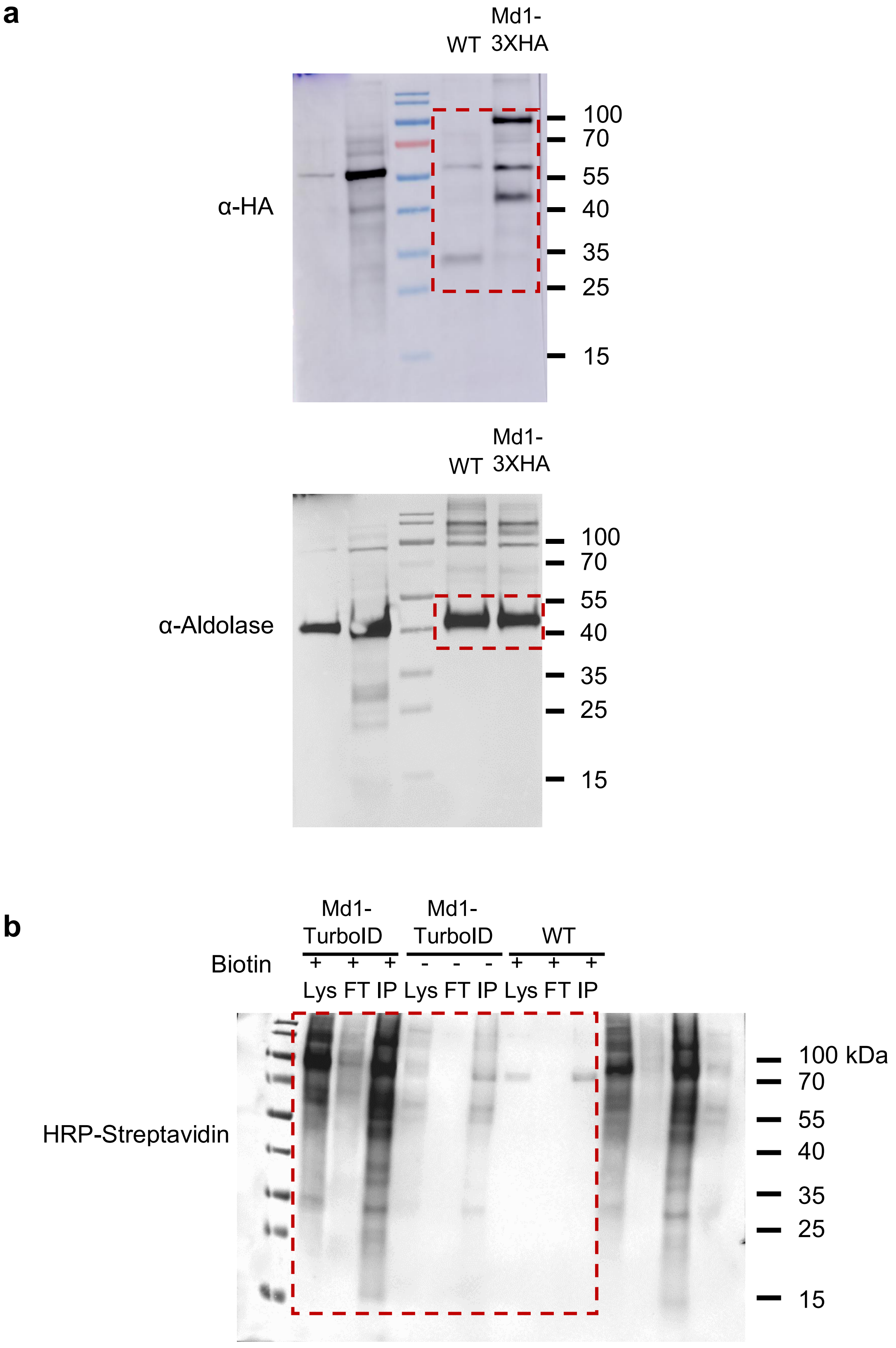

SI Figure S2. Uncropped immunoblots from extended Figure 4. (a) Western blot for extended Data Fig 4c of WT and Md1-3xHA gametocytes fractions. The blot has been probed

with anti-HA, stripped, then probed with anti-aldolase. (**b)** Western blot for extended Data Fig 4g of gametocytes from Md1-TurboID or WT exposed (+) or not (-) to a 2-hour pulse of biotin. Biotinylated proteins were revealed by HRP-streptavidin after streptavidin immunoprecipitation. Lys: input lysate, FT: flowthrough, IP: immunoprecipitated fraction

The following files are deposited in Zenodo and can be accessed here: https://doi.org/10.5281/zenodo.7211710

csv files of processed matrices for single cell RNAseq datasets:

Fig1-10X-data.csv - Count (UMI) table of WT and KO 10X runs with metadata (top 5 rows) used in Figure1.

Fig2-10X-data.csv - Count (UMI) table of WT and Δ270-699 10X runs with metadata (top 5 rows) used in Figure2.

Fig3-counts.csv - Count of 322 Md1-2A-GFP cells used in Figure 3.

Fig3-meta.csv - Metadata of 322 Md1-2A-GFP cells in Figure 3.

Code used in the analysis:

Fig1.R

Fig2.R

Fig3.R

Source data used in figures:

SourceDataFig1.xlsx

SourceDataFig2.xlsx

SourceDataFigED1.xlsx

SourceDataFigED2.xlsx

SourceDataFigED3.xlsx

SourceDataFigED4.xlsx

SourceDataFigED5.xlsx

SourceDataFigED6.xlsx
