# Supplementary material for: A transcriptional switch controls sex determination in Plasmodium falciparum
Source: Nature. 2022 Dec 7;612(7940):528–33. doi: 10.1038/s41586-022-05509-z (PMC9750867; doi:10.1038/s41586-022-05509-z)
Supplement: Supplementary file 2 — Reporting Summary [file 41586_2022_5509_MOESM2_ESM.pdf]

nature portfolio

Corresponding author(s): Arthur Talman

Last updated by author(s): 14/10/22

Reporting Summary

Nature Portfolio wishes to improve the reproducibility of the work that we publish. This form provides structure for consistency and transparency in reporting. For further information on Nature Portfolio policies, see our Editorial Policies and the Editorial Policy Checklist.

Statistics

For all statistical analyses, confirm that the following items are present in the figure legend, table legend, main text, or Methods section.

n/a	Confirmed	
☐	☒	The exact sample size (*n*) for each experimental group/condition, given as a discrete number and unit of measurement
☐	☒	A statement on whether measurements were taken from distinct samples or whether the same sample was measured repeatedly
☐	☒	The statistical test(s) used AND whether they are one- or two-sided<br>*Only common tests should be described solely by name; describe more complex techniques in the Methods section.*
☒	☐	A description of all covariates tested
☐	☒	A description of any assumptions or corrections, such as tests of normality and adjustment for multiple comparisons
☐	☒	A full description of the statistical parameters including central tendency (e.g. means) or other basic estimates (e.g. regression coefficient) AND variation (e.g. standard deviation) or associated estimates of uncertainty (e.g. confidence intervals)
☐	☒	For null hypothesis testing, the test statistic (e.g. *F*, *t*, *r*) with confidence intervals, effect sizes, degrees of freedom and *P* value noted<br>*Give P values as exact values whenever suitable.*
☒	☐	For Bayesian analysis, information on the choice of priors and Markov chain Monte Carlo settings
☐	☒	For hierarchical and complex designs, identification of the appropriate level for tests and full reporting of outcomes
☒	☐	Estimates of effect sizes (e.g. Cohen's *d*, Pearson's *r*), indicating how they were calculated

Our web collection on statistics for biologists contains articles on many of the points above.

Software and code

Policy information about availability of computer code

Data collection

BD FACSDiva software (v 9.0.1) was used during flow cytometry experiments (for both Fortessa and Aria machines).
Imaging acquisition- Zeiss ZEN microscopy software (v2.3 SP1)
Proteomics acquisition- Xcalibur software (v4.2 ThermoFisher Scientific)

Data analysis

Statistical analyses were performed in the R environment (v4.1.0) using RStudio (v 1.4.1717).

The following analysis packages were used during the analysis (specific functions are specified in the methods):
MinKNOW software (version 19.10.1)
Guppy (v3.4.3)
PycoQC (v2.5.0.10)
minimap2 (version 2.17-r941)
Sushi (1.24.1)
Cell Ranger v6.0.1
Trim Gallore (v0.6.5)
HISAT2 (v2.0.0)
HTSeq (v0.12.4)
portcullis (v1.2.2)
bedtools (v2.29.1)
Seurat (v4.0.4)
scDblFinder (v1.6.0)
single cell experiments (v1.8.0)
scater (v1.20.1)
scmap (version 1.8.0)
slingshot (v1.4.0)

MAST (v1.3.4)

Proteomics data analysis
MaxQuant environment (v2.0.3.0)
Leading (v3.4)

Image data analysis
Fiji (v2.3.0) and the GDSC plugin

Cytometry data analysis
FlowJo (v10.7)

Code availibility statement
No new algorithms were developed for this manuscript. Code used for analysis is available in an online repository : https://doi.org/10.5281/zenodo.7211710

For manuscripts utilizing custom algorithms or software that are central to the research but not yet described in published literature, software must be made available to editors and reviewers. We strongly encourage code deposition in a community repository (e.g. GitHub). See the Nature Portfolio guidelines for submitting code & software for further information.

Data

Policy information about availability of data

All manuscripts must include a data availability statement. This statement should provide the following information, where applicable:

- Accession codes, unique identifiers, or web links for publicly available datasets
- A description of any restrictions on data availability
- For clinical datasets or third party data, please ensure that the statement adheres to our policy

Sequencing data that support the findings of this study have been deposited in the European Nucleotide Archive with the accession code PRJEB48349. Processed count data and metadata of single cell data as well as source data for all figures is available in an online repository (https://doi.org/10.5281/zenodo.7211710). The mass spectrometry proteomics data have been uploaded to the ProteomeXchange Consortium via the PRIDE partner repository with the dataset identifiers PXD035547 (Shotgun) and PXD035553 (Interactome). Processed proteomics data is available in Supplementary Table 2.

Field-specific reporting

Please select the one below that is the best fit for your research. If you are not sure, read the appropriate sections before making your selection.

☒ Life sciences ☐ Behavioural & social sciences ☐ Ecological, evolutionary & environmental sciences

For a reference copy of the document with all sections, see nature.com/documents/nr-reporting-summary-flat.pdf

Life sciences study design

All studies must disclose on these points even when the disclosure is negative.

Sample size	Sample size calculation was not performed, minimum sample size for each experiment was determined empirically based on the standards in the field.
Data exclusions	Single cell data was filtered to remove low quality cells based on gene and cell counts.
Replication	All measurements were replicated biologically. The number of biological replicates is stated in each figure legend. Two technical replicates were also performed for qPCR measurements. For knock-out mutants, a mutant strain was complemented, or when that wasn't possible two independent clones of the mutants were generated. Data shown from representative experiments were repeated with similar results in at least 3 independent experiments. All attempts at replication were successful.
Randomization	Our study did not require randomization. Covariates are not relevant to this study.
Blinding	All counting experiments (relative number of females) were blinded by another person than the experimenter before being counted as to avoided experimental bias.

Reporting for specific materials, systems and methods

We require information from authors about some types of materials, experimental systems and methods used in many studies. Here, indicate whether each material, system or method listed is relevant to your study. If you are not sure if a list item applies to your research, read the appropriate section before selecting a response.

Materials & experimental systems

n/a	Involved in the study
☐	☒ Antibodies
☐	☒ Eukaryotic cell lines
☒	☐ Palaeontology and archaeology
☒	☐ Animals and other organisms
☒	☐ Human research participants
☒	☐ Clinical data
☒	☐ Dual use research of concern

Methods

n/a	Involved in the study
☒	☐ ChIP-seq
☐	☒ Flow cytometry
☒	☐ MRI-based neuroimaging

Antibodies

Antibodies used

1- Monoclonal anti-HA antibody produced in Rat; (Roche, 3F10), 11867423001 Sigma, lot: 42155800 (IFA)
2- Monoclonal Anti-α-Tubulin antibody produced in mouse; T5168 Sigma, clone B-5-1-2, ascites fluid; lot : 0000105483
3- Rabbit polyclonal anti-Pfg377 (provided by Pietro Alano) (IFA)
4- anti-HA mouse clone 2-2.2-14 Fisher #11553060 lot #WG327287 (Western)
5- anti-aldolase rabbit polyclonal Abcam #ab38905 lot #GR3242031-4 (Western)
6- anti-mouse HRP polyclonal BioTechne #NBP2-30347H lot #1061068 (Western)
7- goat anti-rat AF488 ThermoFisher #A-11006 lot #2160405 (IFA)
8- goat anti-rabbit AF568 Fisher #10463022 lot #2379475 (IFA)

Validation

1- https://www.sigmaaldrich.com/FR/fr/product/roche/roahaha This antibody has been extensively used in the Plasmodium litterature.
2-https://www.sigmaaldrich.com/deepweb/assets/sigmaaldrich/product/documents/371/375/t5168dat.pdf. The tubulin antibody although it was generated against metazoan tubulin has been extensively used in the literature to label P. falciparum tubulin.
3-The Pfg377 antibody was provided by a colleague, his lab and another independent lab have validated the female specificity of the labelling (Schwank, S., Sutherland, C. J. & Drakeley, C. J. Promiscuous expression of α-tubulin II in maturing male and female Plasmodium falciparum gametocytes. PLoS One 5, e14470 (2010)).
4-https://www.thermofisher.com/order/genome-database/dataSheetPdf?productType=antibody&productsubtype=antibody_primary&productId=26183&version=245
5-https://www.abcam.com/hrp-plasmodium-aldolase-antibody-ab38905.html
6-https://www.novusbio.com/PDFs2/NBP2-30347H.pdf
7-https://www.thermofisher.com/order/genome-database/dataSheetPdf?productType=antibody&productsubtype=antibody_secondary&productId=A-11006&version=245
8-https://assets.fishersci.com/TFS-Assets/LSG/manuals/mp02764.pdf?_ga=2.113207111.2128744841.1665989921-878038475.1658332919

Eukaryotic cell lines

Policy information about cell lines

Cell line source(s)	BEI
Authentication	All cells lines generated in this study were genotyped after initial generation as well as after cloning. Single cell data confirmed the expected genotype of all cell lines tested by this method.
Mycoplasma contamination	Cells lines were not tested for mycoplasma infection
Commonly misidentified lines (See ICLAC register)	There are no commonly misidentified lines in our study

Flow Cytometry

Plots

Confirm that:

☒ The axis labels state the marker and fluorochrome used (e.g. CD4-FITC).

☒ The axis scales are clearly visible. Include numbers along axes only for bottom left plot of group (a 'group' is an analysis of identical markers).

☒ All plots are contour plots with outliers or pseudocolor plots.

☒ A numerical value for number of cells or percentage (with statistics) is provided.

Methodology

Sample preparation	Cultured samples were stained with 2.5 µM of MitoTracker Deep Red FM
Instrument	BD FACsAria II cell sorter - 100 µm nozzle<br>BD LSRFortessa
Software	BD FACSDiva software (v 9.0.1)
Cell population abundance	(a)Viability assay, parasites are 1-5% of the population, viable cells are 30-100 % of cells<br>(b) Parasite (APC positive) represent only a few percent of cells (the rest are uninfected erythrocytes) and with the sorted cells 5-10% were GFP positive.
Gating strategy	(a)Parasites were gated size, single cells and on Vybrant green and the proportion of Mitotracker Deep Red positive event recorded<br>(b)Parasites were gated on size, single cells and APC, and either GFP negative or GFP positive cells were sorted. The index data from the sort is included in the metadata of the single cell RNAseq supplementary file.

☒ Tick this box to confirm that a figure exemplifying the gating strategy is provided in the Supplementary Information.
